# The control mechanisms of heart rate dynamics in a new heart rate nonlinear time series model

**DOI:** 10.1038/s41598-020-61562-6

**Published:** 2020-03-16

**Authors:** Zonglu He

**Affiliations:** Faculty of Management and Economics, Kaetsu University, 2-8-4 Minami-cho, Hanakoganei, Kodaira-shi, Tokyo, 187-8578 Japan

**Keywords:** Computational models, Heart failure, Thyroid gland, Neurophysiology, Statistics

## Abstract

The control mechanisms and implications of heart rate variability (HRV) under the sympathetic (SNS) and parasympathetic nervous system (PNS) modulation remain poorly understood. Here, we establish the HR model/HRV responder using a nonlinear process derived from Newton’s second law in stochastic self-restoring systems through dynamic analysis of physiological properties. We conduct model validation by testing, predictions, simulations, and sensitivity and time-scale analysis. We confirm that the outputs of the HRV responder can be accepted as the real data-generating process. Empirical studies show that the dynamic control mechanism of heart rate is a stable fixed point, rather than a strange attractor or transitions between a fixed point and a limit cycle; HR slope (amplitude) may depend on the ratio of cardiac disturbance or metabolic demand mean (standard deviation) to myocardial electrical resistance (PNS-SNS activity). For example, when metabolic demands remain unchanged, HR amplitude depends on PNS to SNS activity; when autonomic activity remains unchanged, HR amplitude during resting reflects basal metabolism. HR parameter alterations suggest that age-related decreased HRV, ultrareduced HRV in heart failure, and ultraelevated HRV in ST segment alterations refer to age-related decreased basal metabolism, impaired myocardial metabolism, and SNS hyperactivity triggered by myocardial ischemia, respectively.

## Introduction

The autonomic nervous system (ANS) is classically divided into the sympathetic nervous system (SNS) and the parasympathetic nervous system (PNS). The ANS cooperatively modulates heart rate through the two branches. There are ample evidences on how each branch of the ANS operates and modulates heart rate variability (HRV), and the main origins of the low and the high frequency bands under resting conditions^1^. While the unclear role of the ANS remains to be elucidate under other conditions, basically as exercise and/or other increased metabolic demands by the organism as a whole^2^. HRV analysis has become a popular noninvasive tool to assess SNS and PNS activities^1,3^ and is a reliable reflection of the many physiological factors modulating the normal rhythm of the heart^3,4^. However, practical clinical applications of cardiac autonomic tone and cardiovascular risk assessment face complex challenges. For example, HRV exhibits complex nonlinear interactions in healthy individuals, but declines in patients with cardiac diseases such as congestive heart failure^5^ and with age^6,7^. Depression HRV^6,8^ or ultralow and very-low-frequency power of HRV^9^ is strongly associated with a high risk of mortality and cardiac sudden death in individuals with congestive heart failure^9–12^ and diabetic neuropathy^13^. The relations of specific changes in HRV with specific pathologies and aging have not been clarified.

To do this, a large number of studies has been performed to reveal the associations of heart rate variability (HRV) with cardiovascular regulation and autonomic control. The study results include the assessments of the closed-loop interaction between heart period and arterial pressure variabilities and the influence of respiration^14^, the various functional components that comprise the cardiovascular control network by computing transfer functions between nerve stimulation rate and the resulting atrial rate with spectral estimation techniques^15^, and baroreflex gain from spontaneous variability using a causal parametric model^16^; the prediction of blood pressure rhythm based on a baroreflex model of first-order differential-delay equation^17^; the analysis of the influence of time delay in the baroreflex control of the heart activity by using a short-term pressure regulation model^18^; the description of a number of important features of the cardiovascular system including the spontaneous short-term variability in arterial blood pressure and HR data using a beat-to-beat model of the cardiovascular system based on difference equation^19^; an account for oscillations in the blood pressure control system using a nonlinear model that can be related directly to the physiology^20^; quantitative characterization of the physiological mechanisms of fluctuations in heart rate, arterial blood pressure, and instantaneous lung volume using system identification^21^; and an explication of the role of the autonomic regulatory mechanisms in HRV using a mathematical model of short-term cardiovascular regulation^22^.

Heart rate time series exhibit complex behaviors including unpredictability^2^, stochasticity^23^, nonstationarity^1,24^, nonlinearity^4,25^, chaos^25,26^, bifurcation^27^, fractality^6^, and multifractality^28^. Quantifying complexity from heart rate dynamics has attracted a lot of research. For example, Renyi entropy measures of heart rate Gaussianity become a complementary measure of the physiological complexity of the underlying signal transduction processes via robust algorithms^29^; accurate estimation entropy in very short time series is utilized to detect atrial fibrillation in implanted ventricular devices^30^. The use of a resampling procedure along with the size-related correlations of the nonlinear estimator area1 of approximate entropy provides an effective method to discern different generating processes underlying heart rate time series^31^. Another way of solving these challenges is to properly model the generative mechanism of the time series of heart rate (HR) (60/interbeat intervals). Random walk, the simplest case of a nonstationary unit root process that mimics stochastic chaos^32^, with two stochastic feedback controls has successfully modeled the heartbeat regulatory mechanisms and accounted for fractal and nonlinear dynamics of the heartbeat^33^. Nevertheless, the HR model explaining the physiological mechanisms underlying complex heartbeat behavior has not been established.

On the basis of knowledge accumulation in this field, we would like to further clarify the causal relationships of heart rate dynamics with cardiovascular regulation and autonomic control. The aim of this study is twofold. Firstly, we aimed to understand the generative mechanism of the heartbeat time series and the control mechanisms of the stability, patterns, and fractals of heartbeat dynamics. Secondly, we aimed to extract useful information about physiological functions from heart rate data. The key point to achieving the aim is whether the HR model established can pass the validation, that is, whether the outputs of the HR model is acceptable with respect to the real heart rate data-generating process. This also includes considering whether the model has the capability to properly capture the main characteristics of heart rate dynamics. Heart rate is controlled by a stochastic self-restoring mechanism. In recent years, there has been a growing interest in the nonlinear autoregressive integrated (NLARI) process derived by applying Newton’s second law to stochastic self-restoring systems^34–38^. The NLARI process can exhibit the main HR features as mentioned above. For this reason, we adopted the NLARI process to establish the HR model. We validated the HR model by carrying out tests, predictions, simulations, as well as sensitivity and time-scale analysis. Further, this method was applied to detect valuable information from heart rate data and to interpret unsolved problems, which in turn would again validate the HR model.

## Modeling

### NLARI process and model conditions

A system is a stochastic self-restoring system if it sustains (i) a random force or an unpredictable disturbance that may cause a deviation from equilibrium, (ii) a restoring force that reduces the negative (positive) deviation from equilibrium via its upward (downward) component, and (iii) a resistance force that prevents rapid change in response to the perturbations (henceforth NLARI conditions)^34,35^. The self-restoring system can be described by the following NLARI process

1 $$\begin{array}{rcl}{X}_{t} & = & {\theta }_{0}+(1+{\theta }_{1}){X}_{t-1}-{\theta }_{1}{X}_{t-2}+{\theta }_{2}\frac{-({X}_{t-{\kappa }_{2}}-{\mu }_{t-{\kappa }_{2}})}{\exp ({({X}_{t-{\kappa }_{2}}-{\mu }_{t-{\kappa }_{2}})}^{2})}+{\varepsilon }_{t},\\ {\theta }_{0} & = & \omega ,{\theta }_{1}=1-\alpha ,{\theta }_{2}=\beta \ \,{\rm{f}}{\rm{o}}{\rm{r}}\,\ {\kappa }_{1}=1\end{array}$$

where ${\mu }_{t}=E({X}_{t}| {X}_{0},{X}_{-1},\cdots \ ,{X}_{1-{\kappa }_{2}})$ represents the mean for given initial values, *X*_*t*−*j*_ lags *X*_*t*_ by *j* steps at time *t* for *j* = 1, ⋯ , *κ*_2_, ${\mu }_{t}={X}_{0}+(\omega /\alpha )t$ if ${\varepsilon }_{t}={\epsilon }_{t}-E({\epsilon }_{t})$ is Gaussian noise: ${\varepsilon }_{t} \sim N(0,{\sigma }^{2})$, *ϵ*_*t*_ expresses an unpredictable perturbation or noise with mean $\omega =E({\epsilon }_{t})$ and variance ${\sigma }^{2}=var({\epsilon }_{t})$, *α* is the resistance coefficient, *β* is the restoration coefficient, and *κ*_1_ and *κ*_2_ are time lags in resistance and restoration. The relative stable coefficient $\gamma =\beta /(4-2\alpha )$ has been demonstrated to control the stability and bifurcation of the NLARI process^35^. Equation (1) is a nonstationary unit root process lacking the restoring force (*β* = 0). Equation (1) is the deterministic system with a fixed point and a two-period cycle ${(-1)}^{t}\sqrt{{\rm{l}}{\rm{n}}\,\gamma }$ for nonnull initial values and time lags *κ*_1_ = *κ*_2_ = 1 when there is no external disturbance (*σ* = 0). The fixed point is exponentially asymptotically stable if $\gamma \in (0,1)$, while the two-periodic cycle is exponentially asymptotically stable for $\gamma \in (1,\sqrt{e})$ but unstable if $\gamma \in (\sqrt{e},+\infty )$.

Furthermore, the wave indicators *η*_1_ = *ω*∕*α* and *η*_2_ = *σ*∕*β* have been introduced. Due to $$E({X}_{t}| {X}_{0},{X}_{-1})=$$$${X}_{0}+(\omega /\alpha )t$$ when *ε*_*t*_ is Gaussian noise, the ratio *ω*∕*α* represents the slope of the mean line. It has been demonstrated that the ratio *σ*∕*β* is strongly positively correlated with the standard deviation of the data generated by the NLARI process, while the standard derivation of disturbances is a measure of how far the signal fluctuates from the mean. This implies that *η*_2_ = *σ*∕*β* measures the wave amplitude. More importantly, the two wave indicators are also the fractal indicators that determine whether fractal behavior occurs and control the fractal level^36^. Usually, a relatively large absolute slope indicator reflects the high level of dependence, while a relatively small amplitude indicator reflects the high level of self-similarity. The relatively large absolute indicator and the relatively small amplitude indicator can be reached simultaneously by sufficiently aggregating a time series in the stable fixed-point range. That is, typical fractals can be observed if observation scale is large enough (or the frequency of data is low enough) in a self-restoring system. Additionally, a time-delay of an even number in restoration response makes the dependence largely oscillate.

Next, we investigated whether the initiation and propagation of action potential (AP) sustained the three abovementioned forces.

#### Cardiac disturbances and PNS-SNS restoring force

**Figure 1:**
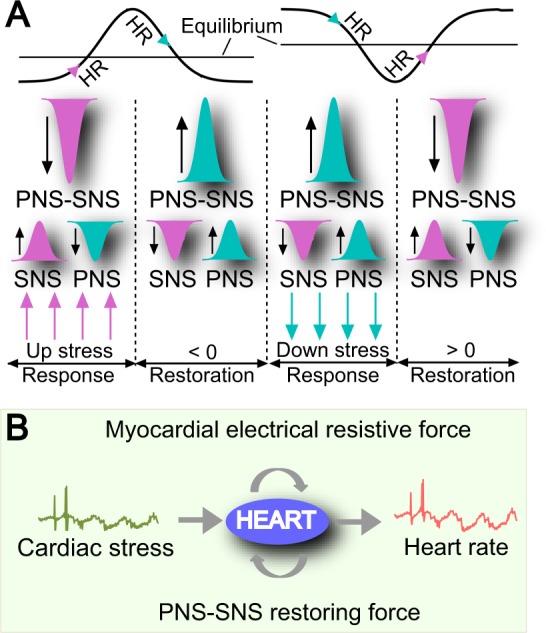
Cardiac sympathetic/parasympathetic nervous system (SNS/PNS) and forces acting on heart rate (HR). (**A**) The relative strength of PNS against SNS activity (PNS-SNS activity or PNS-SNS) played two roles: a response to cardiac stress (noise or stimulus) and a restoring force to maintain HR around equilibrium. (**B**) There were three forces acting on heart rhythms: cardiac stress as a disturbance that may cause heart rate to deviate from the equilibrium heart rate, a restoring force to reduce the derivation via PNS-SNS, and a myocardial electrical resistive force against forward action potential propagation that hinders rapid changes in heart rate.

Heart rate is determined intrinsically by the rate of spontaneous depolarization at the sinoatrial node, but is also modulated by both sympathetic and parasympathetic efferent innervation in response to cardiac disturbances (physical demands, stress, or hormonal factors)^39^. A cardiac disturbance can be driven by an excitatory event, an inhibitory event, or white noise. Excitatory events include acute stress such as low oxygen, high carbon dioxide, ischemia, or hypotension. Inhibitory events include acute stress such as hypertension or certain physiological states such as rest, sleep, comatose, or anesthetic state. Peripheral chemoreceptors located in the aorta, carotid arteries, and the brain are sensory extensions of the peripheral nervous system into blood vessels by which they detect changes in the concentrations of blood borne chemicals and afferent nerves carry them to the brainstem^40^. When baroreceptors located in the carotid sinus and in the aortic arch are excited by a stretch of the blood vessel, they sense the blood pressure changes and relay them to the lower brainstem. The SNS connected to the heart speeds up a slower-than-normal heartbeat by releasing neurohormones known as catecholamines (epinephrine and norepinephrine). The PNS located in the brainstem and upper or sacral portion of the spinal cord slows down a faster-than-normal heartbeat by releasing the neurohormone acetylcholine. The SNS and PNS exerts excitatory and inhibitory effects on target tissue^41^ in regulating processes required for responding to acute stressors and maintaining physiological homeostasis^42^. Homeostasis typically involves negative feedback loops that counteract changes of various properties from their target values^43^. SNS and PNS activities dial heart rate up or down to reduce a negative or positive deviation from the equilibrium heart rate^44^, which provides a negative feedback loop to keep heartbeat homeostasis (Fig. 1A).

#### PNS and SNS modulation

The PNS and SNS work antagonistically, synergistically, or independently to balance the functions of autonomic effector organs^42,45^. Interestingly, the activities of the two branches are not opposites, their interactions are complex^46^, and have a combined effect on HRV and heart rate control in a delicately tuned (see a quite compelling paper: ref. ^47^). The complexity of PNS and SNS modulation is due to the two roles in responding to stimulus and maintaining heart rate in equilibrium. Markedly, there are multiple interactions^48^ and mutual presynaptic inhibition between the SNS and PNS^49,50^. In addition, PNS activation (or deactivation) and SNS deactivation (or activation) have the same impact on heart rate modulation. These results suggest that an inhibitory event activates the PNS, which in turn inhibits the SNS; as a result, PNS activation and SNS deactivation doubly decrease heart rate. Similarly, an excitatory event activates the SNS, which in tun inhibits the PNS; as a result, SNS activation and PNS deactivation doubly increase heart rate. Taken together, the two roles in heart rate regulation can be accomplished more efficiently and effectively by the relative strength of PNS against SNS activity (equivalent to PNS minus SNS activity).

#### Electrical resistance against AP propagation

Cardiac tissues have passive electrical properties such as electric conductivity and electric permittivity of ion channels. Passive electrical properties dominate the electrotonic spread of current through the myocardium and affect AP shape and conduction velocity^51^. The sinoatrial (SA) node cyclically generates AP that passes through the heart via the electrical conduction system causing it to contract. The AP travels across the cell membrane by opening the voltage-gated Na^+^ channels and facilitating the exchange of ions with K^+^, while inhibition of the Na^+^/K^+^-ATPase causes depolarization. The excitation of AP passes through both atria. Then, the atrial depolarization spreads to the atrioventricular (AV) node where the AP slows down just a little due to a physiologically effective refractory period allowing the ventricles time to finish filling with blood. Later, the AP enters through the bundle of His to bundle branches and Purkinje fibers. And lastly, all ventricular muscle becomes activated. AP propagation through the electrical conduction system suffers electrical resistance. Electrical resistivity is the inverse of electric conductivity. A decrease in conductivity or an increase in resistivity can impair AP propagation across the conduction system^52^. Under normal conditions, electrical resistivity is unlikely too large to properly transmit AP, but this does not suggest that AP propagation does not suffer electrical resistance. When electrical resistance is enhanced or altered distinctly due to certain factors such as increased non-uniformity and altered anisotropy caused by structural changes in cardiac disease, normal AP propagation is disturbed, which is a substrate for arrhythmia^53^. Many cardiac arrhythmias are caused by slowed AP conduction, which in turn can be due to an abnormal increase of intracellular myocardial electrical resistance^54^.

### HR models and parameters

The above analysis shows that AP initiation and propagation could meet the NLARI conditions: (i) a cardiac disturbance (noise or stress), to which modulation of PNS versus SNS activity occur in response, potentially causing the heartbeat to deviate from equilibrium; (ii) a restoring force that reduces the positive (negative) deviation from equilibrium by increasing (decreasing) the relative strength of PNS against SNS activity (PNS-SNS activity); and (iii) a myocardial electrical resistive force that hinders a fast change in heart rate (Fig. 1B). For these reasons, we introduced the NLARI process in Eq. (1) to understand the control mechanisms of heart rate dynamics. Notice that ${\mu }_{t}={X}_{0}+(\omega /\alpha )t$ if ${\varepsilon }_{t} \sim N(0,{\sigma }^{2})$. Let ${Y}_{t}={X}_{t}-{X}_{0}-(\omega /\alpha )t$ express the removed-mean/tended HR (henceforth HRV). Equation (1) can be rewritten as

2 $$\begin{array}{l}{Y}_{t}=(1+{\theta }_{1}){Y}_{t-1}-{\theta }_{1}{Y}_{t-2}+{\theta }_{2}\frac{-{Y}_{t-{\kappa }_{2}}}{\exp ({Y}_{t-{\kappa }_{2}}^{2})}+{\varepsilon }_{t}\\ {\theta }_{1}=1-\alpha ,{\theta }_{2}=\beta \ \,{\rm{f}}{\rm{o}}{\rm{r}}\,\ {\kappa }_{1}=1\end{array}$$

The stability involved in homeostasis has never been explicitly stated. Here we define that the NLARI process in the stable fixed-point range is stable homeostasis. The NLARI’s stable fixed point is exponentially asymptotically stable but not globally stable. Formally we introduce the HR models and parameters:

*Definition 1*.  Equation (1) is called the NLARI-HR model (or HR model). Equation (2) is called the NLARI-HRV responder (or HRV responder). The HRV responder in the stable fixed-point range is called the stable homeostatic HRV responder and the noise- or stimulus-driven homeostatic HRV responder for noise or cardiac stress. The HR parameters are assumed to be the physiological indicators as follows:

(1-1) The mean and standard deviation of disturbances *ω* and *σ* are used to measure the mean and standard deviation of myocardial stress, respectively, as the metabolism indicators;
(1-2) The resistance coefficient *α* is used to measure the magnitude of a linear sum of the nonjunctional membrane resistance, the perijunctional resistance, and the junctional resistance belonging to the cells, as the myocardial electrical resistance coefficient;
(1-3) The restoration coefficient *β* is used to measure the relative strength of PNS against SNS activity as the PNS-SNS activity (or autonomic modulation) coefficient;
(1-4) The stability coefficient $\gamma =\beta /(4-2\alpha )$ is used to measure the stability and bifurcation of heart rate dynamics as the HR stability coefficient;
(1-5) The wave indicator *η*_1_ = *ω* ∕*α* (*η*_2_ = *σ*∕*β*) is used to measure HR slope (amplitude) reflecting the relative strength of cardiac stressful level (change) to myocardial electrical resistance (PNS-SNS activity) as the HR slope (amplitude) indicator. Additionally, the wave indicators *η*_1_ and the *η*_2_ are used to measure the HR fractal levels as the HR long-memory indicator and self-similarity indicator, respectively.

**Remark for (1-1)**.  A cardiac disturbance can be driven by physiological or pathological stress. Cardiac stress is triggered by an excitatory event, an inhibitory event, or white noise, depending on whether myocardial metabolic demand exceeds, lowers, or equals delivery of oxygen and nutrients to the myocardium via the coronary circulation. Normal respiratory and metabolic demands cause physiological stress, but changes in respiratory frequency do not affect short-term HRV in healthy populations^55,56^. Thus, the mean and standard deviation of cardiac stress reflect the level and changes in myocardial metabolism if oxygen and nutrient supplies remain unchanged. The basal metabolic rate is controlled by thyroid hormones and also influenced by many factors such as exercise, pregnancy, lack of sleep, gender, age, genetics, body size, weight, and temperature. Instead, the mean and standard deviation of cardiac stress are due to oxygen and nutrient supplies to the myocardium, which may reflect the cardiovascular function, if myocardial metabolism remains unchanged. Pathological stress can be caused by infection, abnormal cardiorespiratory or cardiorespiratory changes, cardiogenic shock, or a chronic infarction scar that may induce monomorphic ventricular tachycardia. Hence, the disturbance mean and standard deviation *ω* and *σ* are expected to reflect myocardial metabolism and cardiorespiratory health.

**Remark for (1-2)**. AP conduction velocity depends on ion channel and physical properties of cardiac myocytes and their interconnections. Impaired conduction can be caused by ion channel defects that alter AP shape, defective coupling between cardiomyocytes, and inherited defects^57^. Hence, the electrical resistance coefficient is expected to reflect the functions of ion channel and physical properties of cardiac myocytes and their interconnections. The resistivity of the gap junction membrane for the passage of ions and small molecules and for propagation is several orders of magnitude higher than the cytoplasmic intracellular resistivity^58^. Gap junction coupling provides a resistance pathway that is several orders of magnitude lower compared with uncoupled membranes^58^, while poor coupling leads to an increase in the gap junction resistance increases during propagation^59^. The perijunctional resistance includes the resistance of the cytolasm within the juctional space, the membrane structure surrounding the junctional lumen, and the resistance of the extracellular fluid outside the junction^60^.

**Remark for (1-3)**. The relative strength of PNS against SNS activity allows the coexistence of PNS activation and SNS deactivation (or SNS activation and PNS deactivation), which doubly decrease (or increase) HR (see Section 2.1.2).

**Remark for (1-4)**. A large stability coefficient can result from a large resistance coefficient, a large restoration coefficient or both (see Section 2.1).

**Remark for (1-5)**. Under the assumption of the HR wave indicators, the fluctuation pattern of heart rate depends on a combined effect of myocardial metabolism, myocardial electrical resistance, or autonomic nervous balance. The ratio of the average myocardial metabolism to myocardial electrical resistance determines the HR slope, while the ratio of myocardial metabolic changes to PNS-SNS activity determines the HR amplitude. When myocardial electrical resistance and autonomic nervous balance remain unchanged, the complexity of heart rate dynamics is due primarily to the sufficiency of myocardial metabolism demand relative to oxygen and nutrient supply. This can explain why physical training increases the complexity of HRV^61^ and why the resting energy expenditure displays significantly positive correlations with heart rate dynamics^62^.

## Model validation

We conducted model validation confirming whether the outputs of the homeostatic HRV responder were acceptable with respect to the real data-generating process by carrying out tests, predictions, and simulations under six conditions.

### Sample description

The data used in this article contained recordings from 30 healthy subjects and 84 patients in the online PhysioNet website which provides well characterized digital heartbeat recordings collected under a variety of conditions^63^. These recordings were divided into six groups: the YOUNG group (young healthy resting wakefulness, 15 subjects, mean age 27 ± 4 years), the ELDER group (elderly healthy resting wakefulness, 15 subjects, mean age 75 ± 5 years), the CHF group (severe congestive heart failure, 15 patients, mean age 56 ± 12 years), the ST group (ST segment alterations with ultrahigh HRV or persistent fluctuations far from equilibrium, 15 patients, mean age 48 ± 16 years), the SCD group (sudden cardiac death, 2 patients, mean age 37 ± 9 years), and the VT/VF group (ventricular tachycardia / ventricular fibrillation, 52 patients, mean age 64 ± 10 years). Each group contained 90 time series. Each time series had 900 points. Each time series with 900 points was used to estimate the HR parameters. See Supplementary Information for more detailed information.

### Statistical tests

We performed statistical tests to identify whether actual heart rate time series followed the homeostatic HRV responder, namely, the NLARI process in the stable fixed-point range. He (2014)^64^ shows the theoretical parameter intervals ${\theta }_{1}\in (-1,1)$, ${\theta }_{2}\in (0,4)$, and $\gamma \in (0,1)$ for the NLARI process within the stable fixed-point range. Moreover, the standard *t* and *F* statistics for testing the theoretical parameter intervals have standard normal limiting distributions when *γ* > 0. This implies that one can adopt the common standard statistical technique to test the homeostatic HRV responder except for *γ* = 0. When the confidence interval does not contain the null hypothesis value, the homeostatic HRV responder is considered statistically significant. For this reason, we examined whether the 95% confidence intervals for *θ*_1_ and *θ*_2_ fell within $(-1,1)$ and $(0,4)$. For testing *γ* = 1, we calculated the *F*-statistic given by

3 $$\begin{array}{ccc}F & = & \frac{RS{S}_{0}-RS{S}_{1}}{RS{S}_{1}/(n-2)}\\ RS{S}_{0} & = & \mathop{\sum }\limits_{t=1}^{n}\,{\left[\Delta ,{Y}_{t},-,\frac{2{Y}_{t-1}}{\exp ({Y}_{t-1}^{2})},-,{\hat{\theta }}_{1},(\Delta {Y}_{t-1}+\frac{2{Y}_{t-1}}{\exp ({Y}_{t-1}^{2})})\right]}^{2}\\ RS{S}_{1} & = & \mathop{\sum }\limits_{t=1}^{n}\,{\left(\Delta ,{Y}_{t},-,{\hat{\theta }}_{1},\Delta ,{Y}_{t-1},+,{\hat{\theta }}_{2},\frac{{Y}_{t-1}}{\exp ({Y}_{t-1}^{2})}\right)}^{2}\end{array}$$

If the *F*-statistic value in Eq. (3) is greater than the 5% (or 1%) critical value of an *F*distribution with 1 numerator degrees of freedom and *n* − 2 denominator degrees of freedom, then the null hypothesis *γ* = 1 should be rejected. For *γ* = 0, we performed the *γ*_*n*_ statistic test where the *γ*_*n*_ statistic is given by

4 $${\gamma }_{n}=4\sqrt{\pi n{(32{\widehat{\sigma }}^{6})}^{-1}}\sqrt{1-{\widehat{\theta }}_{1}}{\widehat{\theta }}_{2}$$

The statistic has a special limiting distribution $W(1)/\sqrt{L(1,0)}$ under the given assumptions. Table 1 in ref. ^64^ provides the critical values for the *γ*_*n*_ statistic.

**Figure 2:**
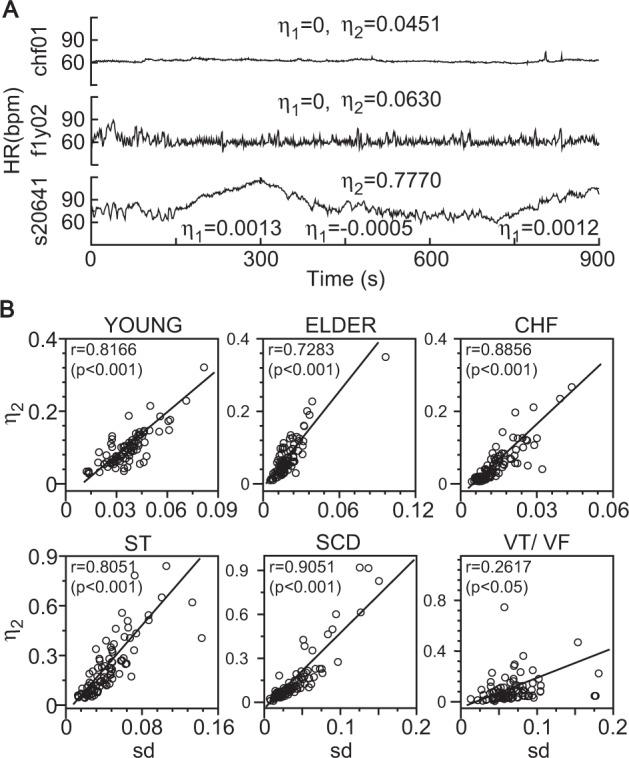
Heart rate (HR) slope and amplitude indicators *η*_1_ and *η*_2_. (**A**) HR for chf01, f1y02, and s20641. *η*_1_ = 0 (chf01, f1y02) corresponded to a horizontal mean line, a slightly larger absolute slope indicator corresponded to a slightly larger slope of the mean heartbeat line (s20641) (an upward-trending mean line for *η*_1_ = 0.0013: 131–300; a downward-trending mean line for *η*_1_ = −0.0005: 301–700; and an upward-trending mean line for *η*_1_ = 0.0012: 701–900). A slightly larger amplitude indicator corresponded to a larger amplitude (*η*_2_ = 0.0451 for chf01, *η*_2_ = 0.0630 for f1y02; *η*_2_ = 0.1722 for s20641:1–900 points). (**B)** Scatter plots showing a strong positive correlation between the sample standard deviation from the mean *sd* and estimated *η*_2_ for the YOUNG and ELDER groups (no cardiac diseases), CHF (congestive heart failure), ST (ST segment alteration), SCD (sudden cardiac death), and VT/VF (ventricular tachycardia/ventricular fibrillation) groups.

Table 1 shows that all the parameter values fell within the theoretical parameter intervals of the homeostatic HRV responder ${\theta }_{1}\in (-1,1)$, *θ*_2_ ∈ (0, 4), and *γ* ∈ (0, 1). All the minimums ${\widehat{\theta }}_{i,\min }$ of the lower boundaries and all the maximums ${\widehat{\theta }}_{i,\max }$ (*i* = 1, 2) of the upper boundaries for the two-sided 95% confidence intervals were situated within (−1, 1) for *θ*_1_ and (0, 4) for *θ*_2_ (90% for 0159.vf1) where ${\widehat{\theta }}_{1}$ and ${\widehat{\theta }}_{2}$ were the Ordinary Least Squares (OLS) estimates of *θ*_1_ and *θ*_2_. Thus, the data support *θ*_1_ ∈ (−1, 1) and *θ*_2_ ∈ (0, 4). All the *F-*test for the hypothesis *γ* = 1 were strongly rejected. Moreover, all the minimums ${\gamma }_{{\rm{n}},\min }$ of the statistic $| {\gamma }_{n}|$ for testing *γ* = 0 were far above 11.9 at the 99% significance level for sample sizes ranging from *n* = 900 to 10000 in the six groups. Thus, the hypothesis *γ* = 0 was rejected at the 1% significance level. These two lines of evidence support the hypothesis $\gamma \in (0,1)$. Thus, the data support that the homeostatic HRV responder was the heartbeat data generative process. This implied that the dynamic control mechanism of heart rate was a stable fixed point rather than a fast transition between a fixed point and limit cycle or chaos. Furthermore, Fig. 2A shows that a larger $| {\eta }_{1}|$ value corresponded to a steeper slope of the mean heartbeat line, while a larger *η*_2_ value corresponded to a higher HR amplitude. Figure 2B indicates that there was a significant positive correlation between the estimated *η*_2_ and the sample *sd*, which supported that *η*_2_ was the amplitude indicator. These results showed that the wave indicators *η*_1_ and *η*_2_ had good performances in measuring the HR slope and the HR amplitude.

**Table 1:** Test results for the homeostatic HRV responder.

Group	${\gamma }_{{\rm{n}},\min }$	${\widehat{\theta }}_{1,\min }$	${\widehat{\theta }}_{1,\max }$	${\widehat{\theta }}_{2,\min }$	${\widehat{\theta }}_{2,\max }$
YOUNG	49.45	−0.5695	0.5924	0.0485	0.8759
ELDER	58.99	−0.4119	0.4110	0.0222	0.7557
CHF	139.11	−0.5870	0.2606	0.0075	1.1335
ST	31.37	−0.6229	0.4507	0.0028	0.4299
SCD	17.37	−0.6770	0.2255	0.0106	0.7230
VT/VF	34.76	−0.6614	0.7390	0.0011	2.3028

${\gamma }_{n,\min }$ is the minimum of $| {\gamma }_{n}| ,{\gamma }_{n}$ is the statistic to test for $\gamma =0,{\widehat{\theta }}_{1,\min }\,({\widehat{\theta }}_{1,\max })$ and ${\widehat{\theta }}_{2,\min }({\widehat{\theta }}_{2,\max })$ are the minimums (maximums) of low (upper) boundaries at 95% confidence intervals of *θ*_1_ and *θ*_2_.

### Predictions

**Figure 3:**
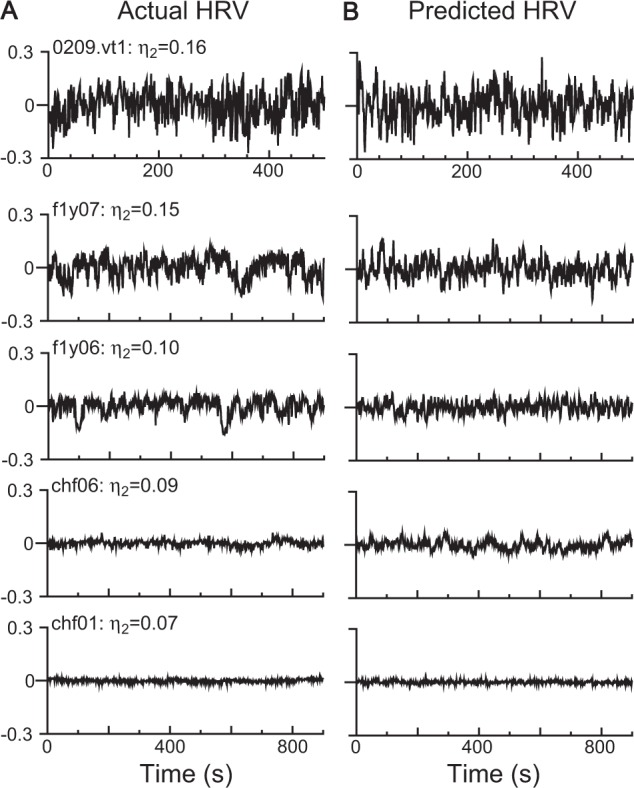
Prediction performance of the noise-driven HRV responder. The HRV responder within the stable fixed-point range is called the homeostatic HRV responder. The predicted traces by the noise-driven homeostatic HRV responder (**B)** mimicked real HRV traces for 0209.vt1 (ventricular tachycardia), f1y07 and f1y06 (young healthy), and chf06 and chf01 (congestive heart failure). (**A**) Moreover, the amplitude of HRV decreased as the amplitude indicator *η*_2_ decreased.

A good out-of-sample forecast performance provides strong evidence for the data generative process. To determine whether the homeostatic HRV responder has a good out-of-sample forecasting performance, the HRV responder was used to predict HRV dynamics under six groups. We discovered for the first time that the noise-driven homeostatic HRV responder predicted most of the HRV time series in the YOUNG, ELDER, and CHF groups and some HRV time series in the VT/VF group. For example, the homeostatic HRV responder (Fig. 3B) based on in-sample data exhibited similar patterns as the original ones drawn by out-sample data (Fig. 3A) where a lower *η*_2_ value resulted in a lower HRV amplitude, which provides evidence that *η*_2_ is an amplitude indicator. However, the noise-driven homeostatic HRV responder could not predict HRV trajectories driven by unpredictable pathological stress.

**Figure 4:**
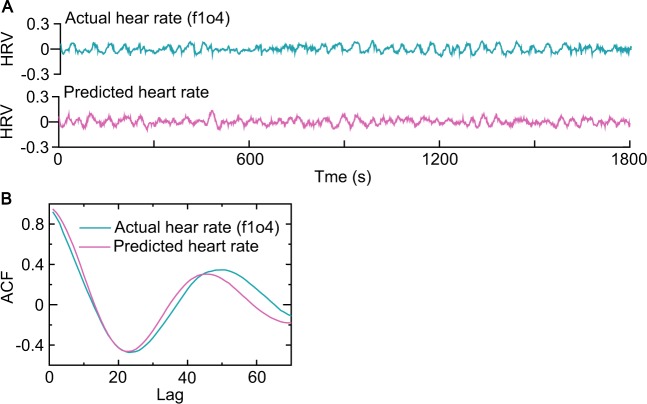
Predictions of HR dynamics and long memory. (**A)** The out-of-sample predicted HRV trace exhibited similar trace to the actual HRV from an elderly subject (f1o04: 1801–3600 points). (**B)** The out-of-sample predicted ACF trace better mimicked the actual ACF trace based on the heartbeat data from the same subject (f1o04: 1801–3600 points) with a restoration delay of 10. The predicted ACF values were calculated using the data generated by the noise-driven homeostatic HRV responder as a realization of the random variable. The predicted and actual HRV responders in (**A**,**B**) had the same parameter values estimated using the heartbeat data from the same subject (f1o04: 1–1800 points). A process has long memory if its autocorrelation function (ACF) decays more slowly than an exponential decay (e.g., at a hyperbolic rate or an oscillation) as the lag increases. These results show the ability of the noise-driven homeostatic HRV responder to predict the HRV dynamics and oscillatory long memory for a long even restoration delay.

Furthermore, Fig. 4 shows that the noise-driven homeostatic HRV responder successfully predicted not only HRV dynamics (Fig. 4A) but also a long memory for a restoration delay of 10 (Fig. 4B) (a process has long-range dependence if its autocorrelation function (ACF) decays more slowly than an exponential decay as the lag increases). This result confirmed the finding that a time delay of an even number in restoration could lead to long-term large fluctuations^36^.

### Simulations

**Figure 5:**
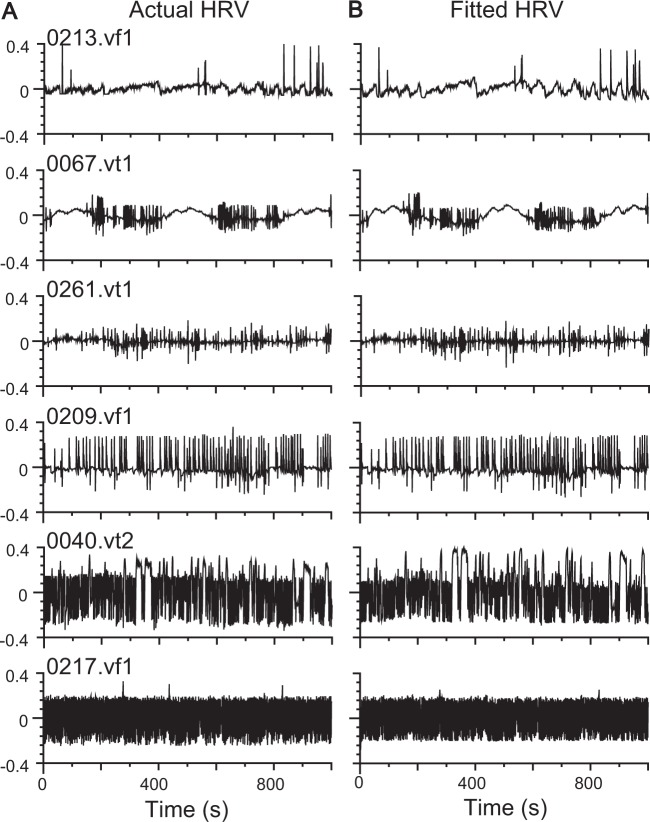
Fitting performance of the stimulus-driven homeostatic HRV responder. (**A)** The real HRV trace plots of 0213.vf1 (ventricular fibrillation), 0067.vt1 (ventricular tachycardia), 0261.vt1 (ventricular tachycardia), 0209.vf1 (ventricular fibrillation), 0040.vt2 (ventricular tachycardia), and 0217.vf1 (ventricular fibrillation). (**B**) The fitted traces produced by the stimulus-driven homeostatic HRV responder. These fitted traces mimic real HRV, implying that the stimulus-driven homeostatic HRV responder can describe pathological arrhythmias when cardiovascular perturbations are unpredictably pathological stimuli or intense vibration. These arrhythmias reflect the pathological state of cardiovascular and autonomic nervous systems.

We examined the simulation performance of the homeostatic HRV responder under six conditions (groups). We discovered that the noise-driven homeostatic HRV responder failed to simulate HRV situated at the edge of stability (a near zero stability coefficient) in a subset of ST, SCD, and CHF groups. Instead, the stimulus-driven homeostatic HRV responder could accurately simulate most of the HRV time series in the VT/VF group as shown in Fig. 5.

As shown above, the homeostatic HRV responder could have a good predictive and simulation performance for many HRV time series. Notably, the estimated parameters may be inconsistent if the stability coefficient is close to 1. In this case, the parameter value can be approximated to achieve a good fit by carrying out simulation as shown in 0217.vf1 in Fig. 5 (see Supplementary Information).

### Sensitivity analysis

The stability of the NLARI-HR model results from the impact of uncertainties on the performance of HR parameter estimates. Sources of uncertainty in HR parameter estimates are due primarily to the randomness of heartbeat samples and changes in their inputs. In Section 3.2, we demonstrate that almost all heartbeat time series data lie within the stable fixed-point range at a 95% confidence interval showing that the effect of uncertainty caused by randomness on HR parameters $(\alpha ,\beta ,\gamma )$ was small. Thus, we focus here on parameter sensitivity analysis to quantify uncertainty and the propagation of uncertainty caused by changes in the inputs when encountering unexpected relationships between inputs and outputs. Sensitivity is usually measured by the ratio of the percentage change in an output (or a dependent variable) to the percentage change in an input (or an independent variable). Here we consider the sensitivity of HR parameters in Eq. (1) to a sequence of unexpected small random perturbations ${\epsilon }_{it}\,{\rm{{\prime} }}$ with mean *ω*_*i*_ and variance ${\sigma }_{i}^{2}$, where ${\varepsilon }_{it}{\rm{{\prime} }}={\epsilon }_{it}{\rm{{\prime} }}-{\omega }_{i}$ is a white noise independent of the white noise *ε*_*t*_ for *i* = 1, ⋯ , *n*. The disturbed NLARI process is given by

5 $${X}_{t}=(\omega +{\omega }_{i})+(2-\alpha ){X}_{t-1}-{(1-\alpha )}_{t-2}+\beta \frac{-({X}_{t-2}-{\mu }_{t-2})}{\exp {({X}_{t-2}-{\mu }_{t-2})}^{2}}+({\varepsilon }_{t}+{\varepsilon }_{it}{\prime} )$$

where ${\mu }_{t}=[(\omega +{\omega }_{i})/\alpha ]t$. The sensitivity of HR parameters to changes in the inputs can be calculated using the following formula:

*Definition 2*.  The sensitivity of HR parameter *ϕ* = *α*, *β*, *γ*, *σ*, *η*_2_ to changes in inputs Δ*φ*_*i*_ = Δ*ω*_*i*_ or *ϕ* = *ω*, *α*, *β*, *γ*, *η*_1_ to changes in inputs *Δφ*_*i*_ = *Δσ*_*i*_ is given by

6 $$S({\varphi }_{i},\phi )={{\rm{l}}{\rm{i}}{\rm{m}}}_{\Delta {\varphi }_{i}\to 0}\left|\frac{\Delta {\phi }_{i}/\phi }{\Delta {\varphi }_{i}/\varphi }\right|\approx \left|\frac{\Delta {\phi }_{i}/\phi }{\Delta {\varphi }_{i}/\varphi }\right|$$

for *i* = 1, ⋯ , *n*. We say that the HR parameter *ϕ* relative to the perturbations Δ*φ*_*i*_ is perfectly insensitive if $S({\varphi }_{i},\phi )=0$, insensitive if $S({\varphi }_{i},\phi ) < 1$, unit sensitive if $S({\varphi }_{i},\phi )=1$, and sensitive if $S({\varphi }_{i},\phi ) > 1$.

**Figure 6:**
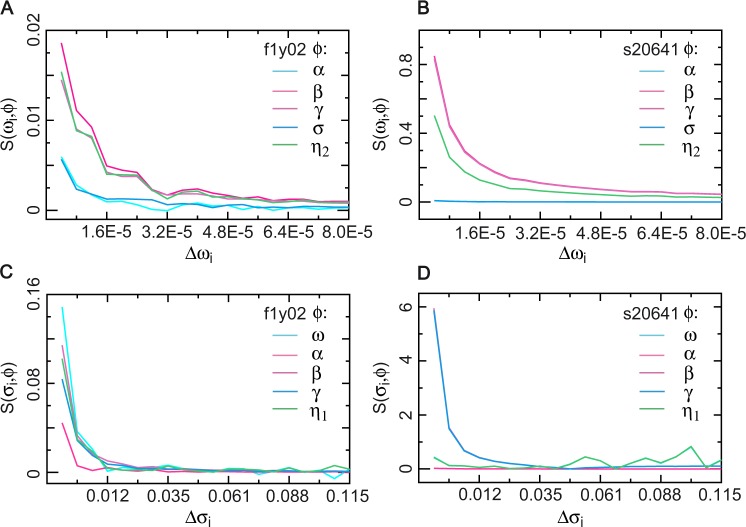
Sensitivity of HR parameters. (**A**,**B**) The sensitivity $S(\omega ,\phi )$ of the HR parameter *ϕ* = *α*, *β*, *γ*, *σ*, *η*_2_ to changes in the mean of unexpected perturbation Δ*ω*_*i*_ (*i* = 1, ⋯ , 20) based on the HR parameter estimates of heart rate time series f1y02 and s20641. (**C**,**D**) The sensitivity $S(\sigma ,\phi )$ of *ω*, *α*, *β*, *γ*, *η*_1_ to changes in the standard deviation of unexpected perturbation Δ*σ*_*i*_ for f1y02 and s20641. Heart rate time series f1y02 (900 samples) followed the NLARI process within the stable fixed-point range, while heart rate time series s20641 (900 samples) followed the NLARI process on the boundary of the stable fixed-point range.

Due to $Var({\varepsilon }_{t}+{\varepsilon }_{it}{\prime} )={\sigma }^{2}+{\sigma }_{i}^{2}$, $\Delta {\sigma }_{i}=\sqrt{{\sigma }^{2}+{\sigma }_{i}^{2}}-\sigma$. Note that $\Delta {\omega }_{i}=(\omega +{\omega }_{i})-\omega ={\omega }_{i}$. Then, we have $S({\omega }_{i},\phi )=| ({\phi }_{i}-\phi )\omega /({\omega }_{i}\phi )|$ and $S({\sigma }_{i},\phi )=| ({\phi }_{i}-\phi )\sigma /[(\sqrt{{\sigma }^{2}+{\sigma }_{i}^{2}}-\sigma )\phi ]|$. We conducted simulations to calculate the sensitivity $S({\omega }_{i},\phi )$ and $S({\sigma }_{i},\phi )$ based on the HR parameter estimates of f1y02 from the YOUNG group and s20641 from the ST group, which represented a stationary (the stability coefficient *γ* = 0.1787 for f1y02, 900 samples) and a nonstationary (*γ* = 0.0171 for s20641, 900 samples) heart rate time series, respectively. The sensitivity of the average estimation of HR parameters over 3000 repeated simulations is presented in Fig. 6A,B for *ϕ* = *α*, *β*, *γ*, *σ*, *η*_2_ to changes in the perturbations Δ*ω*_*i*_ and in Fig. 6C,D for *ϕ* = *ω*, *α*, *β*, *γ*, *η*_1_ to changes in the perturbations Δ*σ*_*i*_ for f1y02 and s20641, where *ω*_*i*_ = 0.00004*i* and *σ*_*i*_ = 0.007*i* for *i* = 1, ⋯ , 20. Therefore, the parameter sensitivity analysis indicates that the HR parameters were insensitive or perfectly insensitive for the NLARI process within the stable fixed-point range, whereas the restoration coefficient and the stability coefficient relative to changes in the standard deviation of perturbations were sensitive for the NLARI process on the boundary of the stable fixed-point range.

### Time-scale analysis

**Figure 7:**
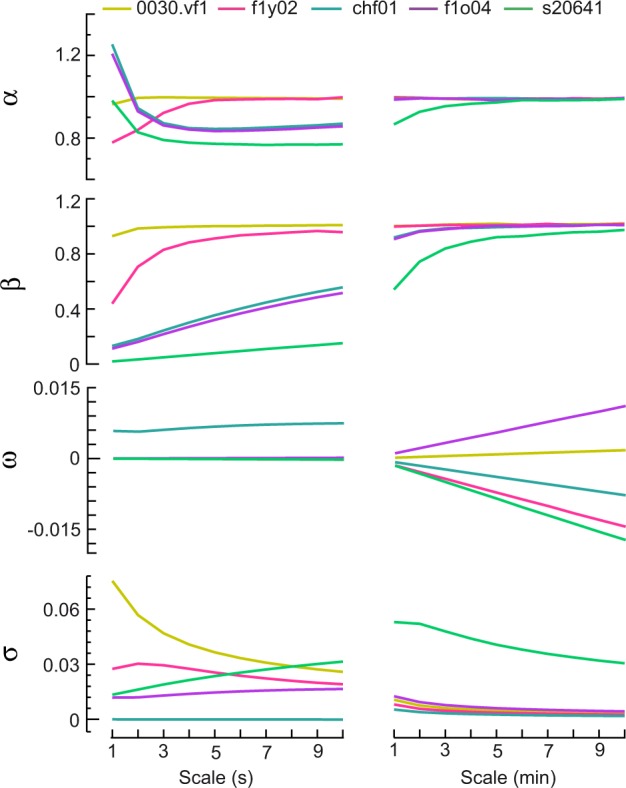
Time scales covered by the HR model. The polylines on the left- and right-hand side reflect changes in HR parameter estimates on average at the second scales *m* = 1, ⋯ , 10 (s) and the minute scales *m* = 1, ⋯ , 10 (min), respectively, where *α* is the myocardial electrical resistance coefficient, *β* is the PNS-SNS activity coefficient, *ω* is the the mean of metabolic perturbations, and *σ* is the standard deviation of metabolic perturbations. The figure below presents HR parameter values of *m* = 1 for f1y02, f1o04, chf01, s20641, and 0030.vf1 (900 samples).

The heartbeat time scales or observation scales in the literature range from a few seconds (describing oscillations in the respiratory rate) to minutes (describing oscillations linked to vasomotion and modifications to peripheral resistances). Thus, it is necessary to perform time scale analysis to clarify the suitable time scale covered by the NLARI-HR model. That is, we need to assess the effects of different time scales on HR parameter estimates. Enlarging the time scale of a time series can be realized by aggregating the time series using ${Y}_{t}^{(m)}=\frac{1}{m}{\sum }_{i=1}^{m}{Y}_{(t-1)m+i}$ for $t=1,\cdots \ ,[T/m]$ ($[T/m]$ denotes the integer part of *T*∕*m*)^36^. Consider the observation scales ranging from 1 to 10 seconds and from 1 to 10 minutes and real f1y02, f1o04, chf01, s20641, and 0030.vf1 (which were regarded as the representative heart rate time series of the YOUNG, ELDER, CHF, ST, and VT/VF groups, respectively) on the first scale *m* = 1. We conducted simulations to estimate the basic HR parameters $(\alpha ,\beta ,\omega ,\sigma )$ and obtained the average HR parameter values over 3000 repeated simulations. From Fig. 7, we see that as the time scale increases, the myocardial electrical resistance coefficient (*α*) and PNS-SNS activity coefficient (*β*) converged to the same value of 1 on the minute scale, in which the larger the PNS-SNS activity coefficient was, the faster the velocity of convergence (e.g., s20641, with the smallest value *β* = 0.0171, had the slowest convergence speed, while 0030.vf1, with the largest value *β* = 0.9274, had the fastest convergence speed). The standard deviation of metabolic perturbations (*σ*) converged to a small value, whereas the mean of metabolic disturbances (*ω*), except for 0030.vf1, which had a small value, was continuously enlarged at the minute scale. These results indicate that HR parameters on the minute scale cannot exactly extract physiological information from heart rate data because of ultra-enlarged scales.

### Comparison with parametric power spectral analysis

**Figure 8:**
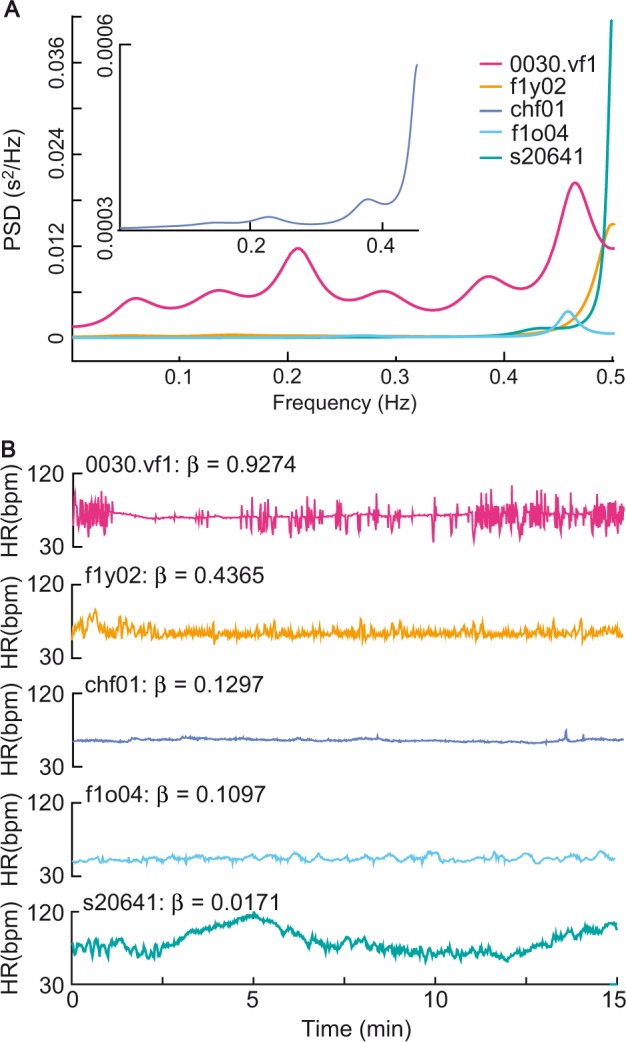
Comparison of the restoration coefficient with the PSD in actual data. (**A**) The power spectral density (PSD) of 0030.vf1, f1y02, chf01, f1o04, and s20641 from the VT/VF, YOUNG, CHF, ELDER, and ST groups. (**B**) HR trajectories and the restoration coefficient (*β*) for 0030.vf1, f1y02, chf01, f1o04, and s20641. The restoration coefficient estimates were roughly consistent with their PSDs for f1y02, chf01, and f1o04, but significantly inconsistent with the PSD for s20641. Heart rate time series s20641 exhibited very low-frequency fluctuations, which was inconsistent with its very low LF/HF ratio but consistent with its small restoration coefficient.

It is important to compare the performance of the present method with the classical parametric power spectral analysis in assessing the function of the autonomic nervous system. The power spectral density analysis of HR time series in the very low-frequency (VLF: 0 to 0.05 Hz), low-frequency (LF: 0.05 to 0.15 Hz), and high-frequency (HF: 0.15 to 1.0 Hz) bands provides a quantitative noninvasive tool to assess the ANS modulation. It is widely accepted that a high LF means an increase in the activity of the SNS, while a high HF shows an increase in the activity of the PNS. Thus, the ratio of LF to HF power (LF/HF ratio) is applied to estimate the ratio between SNS and PNS activity^3,65^. The restoration coefficient was regarded as the PNS-SNS activity metric. Here we compared the restoration coefficient (*β*) with the parametric power spectral density (PSD) for different frequency bands, obtained by means of the autoregressive model of order *p* = 16. The restoration coefficient estimates and their PSDs were roughly consistent for some heart rate data (such as f1y02, chf01, and f1o04 in Fig. 8), but significantly inconsistent with its PSD for some heart rate data (such as 0030.vf1 in Fig. 8). For example, in the four heart rate time series, s20641 had the lowest LF/HF ratio, which implied that its PNS activity was far greater than its SNS activity; on the other hand, it exhibited large fluctuations away from the average, with a very low-frequency showing that its PNS activity was far less than its SNS activity. This contrariety did not occur for the restoration coefficient to measure PNS-SNS activity. For example, s20641, which had a small restoration coefficient, was consistent with large fluctuations away from the average value showing a weak PNS to SNS activity.

**Figure 9:**
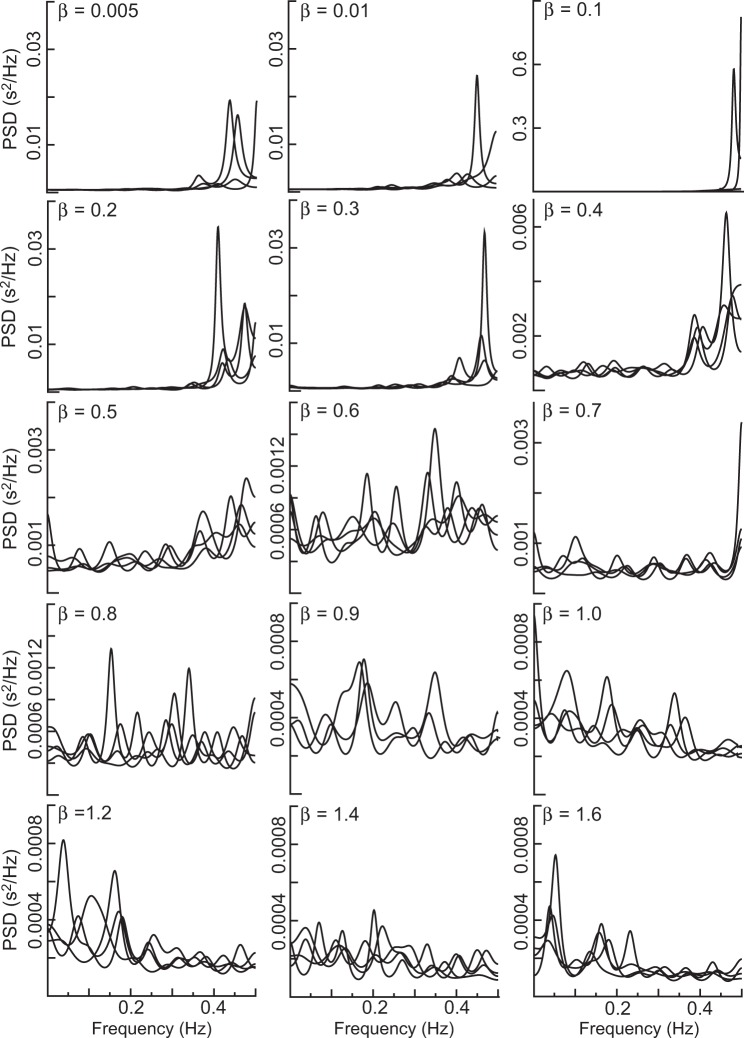
Comparison of the restoration coefficient with the PSD in simulation data. The realizations of the PSD were obtained by the autoregressive model of order *p* = 16 at the time scale of *m* = 3 for the restoration coefficient *β* from 0.005 to 1.6. The original time series (*m* = 1) were generated by the NLARI process in Eq. (2), where the time delay *κ*_2_ = 1, the resistance coefficient *α* = 0.7786, the standard deviation *σ* = 0.0275, and the sample size *T* = 1500. It is seen that a small restoration coefficient $\beta \in [0.01,0.1]$ could correspond to a very low LF/HF ratio (s20641 with *β* = 0.0171); a relatively large restoration coefficient $\beta \in (0.1,0.5)$ could have a low LF/HF ratio (f1o04 with *β* = 0.4365), a relatively low LF/HF ratio (f1o04 with *β* = 0.1097), a relatively high LF/HF ratio (chf01 with *β* = 0.1297); and a large restoration coefficient $\beta \in [0.6,1.6]$ could trend to a high LF/HF ratio (0030.vf1 with *β* = 0.9274).

To illustrate the phenomenon observed in Fig. 8, we performed simulation study to illustrate the phenomenon observed in Fig. 8 and found that PSD estimation was very nonstationary for a nonstationary, a nonlinear stationary, or a nonlinear nonstationary time series. The simulation result confirmed that the classical parametric power spectral analysis was not suitable for nonstationary and nonlinear stochastic processes. We note that an adaptive observation scale could reduce the nonstationarity. Then, simulations were carried out at the observation scale of *m* = 3 to estimate the PSD under different restoration coefficients. Figure 9 presents four realizations of the PSD for each case using the time series generated by the NLARI process in Eq. (2). It is seen that as the restoration coefficient increases, the LF/HF ratio tends to (i) decrease for 0 < *β* ≤ 0.1 (which was consistent with the observation that s20641 had a very low LF/HF ratio in *β* = 0.0171 in Fig. 8), (ii) decrease or increase for 0.2 ≤ *β* ≤ 0.4 (which was consistent with the observation that the restoration coefficients of f1o04, chf01, and f1y02 roughly corresponded to their LF/HF ratios in Fig. 8), and (iii) increase for 0.5 ≤ *β* ≤ 1.6 (which was consistent with the fact that 0030.vf1 had a large LF/HF ratio for a large *β* = 0.9274 in Fig. 8). The PSD realizations show the possibility that in a relatively stable fixed-point range, a low LF/HF ratio reflected a high PNS. However, contrary to previous expectations, a high LF/HF ratio might be due to overactive PNS rather than overactive SNS.

**Figure 10:**
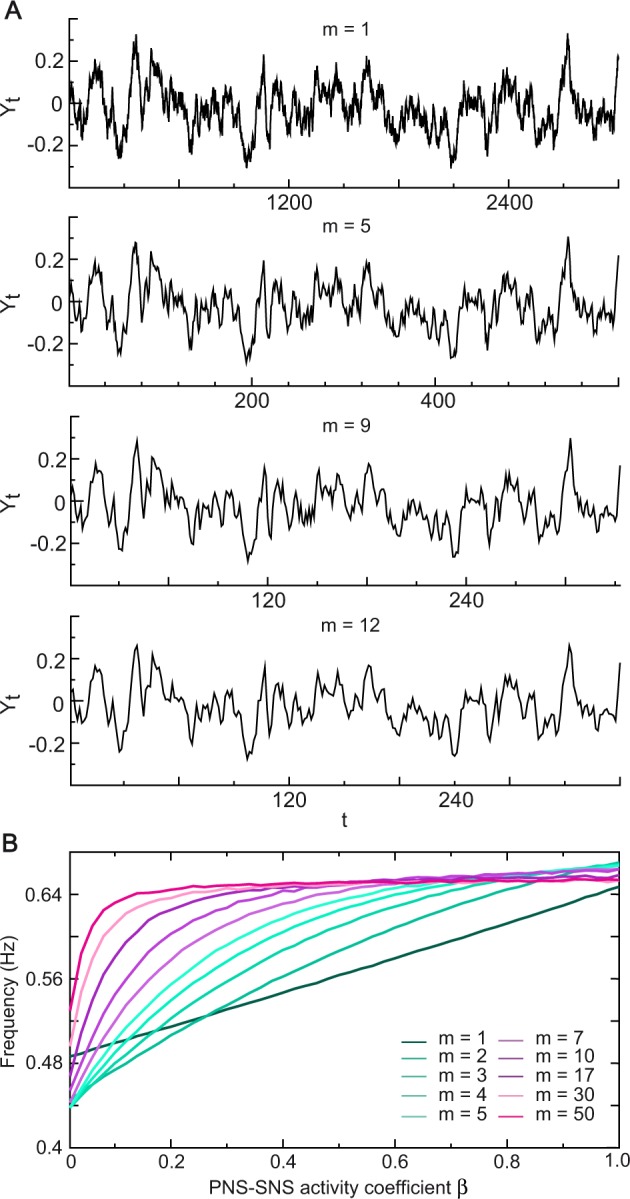
Relationship between the frequencies and the restoration coefficient. (**A**) The trajectories of the time series generated by the NLARI process on different time scales ranging from *m* = 1 to 50. As the time scale increases, the trajectories of the time series became more smooth. (**B**) A plot of frequency of the time series against the restoration coefficient *β*. The time series were generated by the NLARI process in Eq. (2) using the resistance coefficient *α* = 0.9489, *β* = 0.02*i* for *i* = 1, ⋯ , 50, the standard deviation *σ* = 0.022, the sample size 3000, and repeated times 3000. It is shown that the frequency of the time series was positively related to the restoration coefficient, but the frequency became insensitive to an increased restoration coefficient for an over enlarged time scale.

To explore whether there is the relation between the frequencies and the restoration coefficient, it is necessary to remove the impacts of randomness and nonstationarity of HR time series on PSD estimation. We performed simulations to estimate the average frequency over 3000 repeated times. The frequency of a time series that exhibits irregular variations changes for different time scales (Fig. 10A). For this reason, we examined whether there was a relationship between the frequencies and the restoration coefficient at observation scales ranging from *m* = 1 to 50. Figure 10B shows a positive correlation between the frequency and the restoration coefficient, but the positive correlation did not exist in low-frequency bands, which was consistent with the observation that high LF/HF ratio did not certainly represent a high SNS activity in Fig. 9. Additionally, we note that the frequency became insensitive to an increased restoration coefficient on an over high time scale.

## Applications of HR parameters

**Figure 11:**
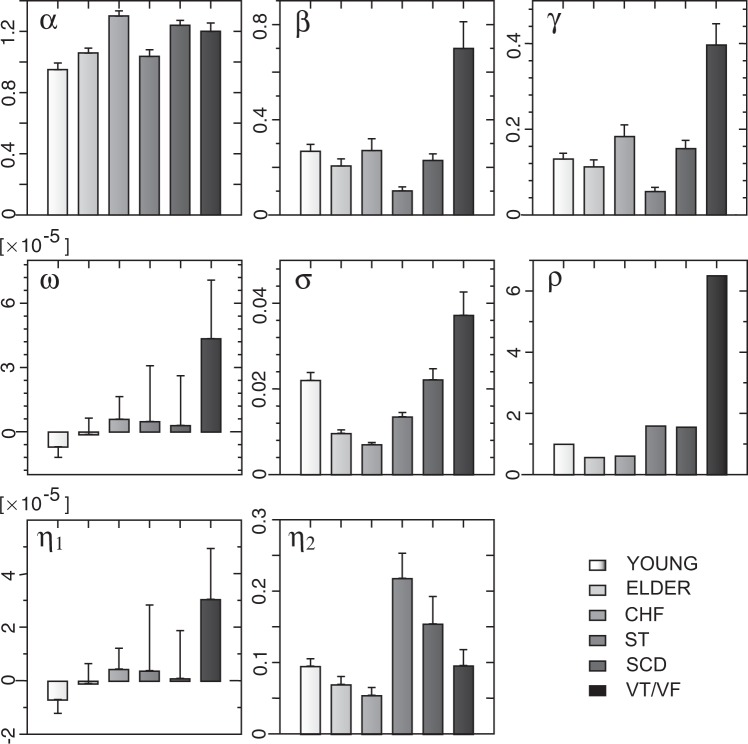
HR parameter alterations. Myocardial electrical resistance indicator *α* had an increase in the ELDER group, the largest increase in the CHF group, an ultraincrease in the SCD and VT/VF groups. PNS-SNS activity indicator *β* had a decrease in the ELDER group, a mild decrease compared to age in the CHF group, the largest decrease in the ST group, and the largest increase in the VT/VF group. HRV stability indicator *γ* had a decrease due to decreased PNS-SNS activity in the ELDER group, an overt increase due to ultraincreased myocardial resistance in the CHF group, the largest decrease due to ultradecreased PNS-NS activity in the ST group, and the largest increase due to ultraincreased myocardial resistance/PNS-SNS activity in the VT/VF group. The mean in myocardial metabolic rate *ω* had the largest increase in the VT/VF group leading to the largest increase HR amplitude indicator *η*_1_. The sd in myocardial metabolic rate *σ* had an ultradecrease in the EDLER group, the largest decrease in the CHF group, and the largest increase in the VT/VF group. HR amplitude indicator *η*_2_ had a decrease due to ultradecreased changes in myocardial metabolic rate in the ELDER group, the largest decrease due to mildly increased PNS-SNS activity and ultradecreased changes in myocardial metabolic rate in the CHF group, the largest increase due to the lowest changes in myocardial metabolic rate in the ST group, and an ultraincrease in the SCD group. The risk predictor in the SCD group had the lowest values in the ELDER and CHF groups, the second high value in the ST and SCD groups, and 6-fold increase in the VT/VF group.

We compared the HR parameter estimates in five groups with those in healthy young group. Figure 11 showed the HR parameter alterations in the five conditions on the group-average estimate. We explored the possible relations of HR parameter alterations to cardiac, metabolic, and autonomic nervous functions based on physiological knowledge.

### Young versus elder subjects

Comparison between the HR parameter estimates in the YOUNG and ELDER groups showed that healthy older subjects at rest had increased myocardial electrical resistance coefficient (*α*↑), decreased PNS-SNS activity coefficient (*β*↓), a near zero average value of myocardial metabolism (− *ω*↓0) leading primarily to a near zero HR slope indicator (− *η*_1_↓0), and an ultradepressed standard deviation of myocardial metabolism (*σ* ⇊ ) leading primarily to decreased HR amplitude indicator (*η*_2_↓). These results suggested that aging was related to increased myocardial electrical resistance, decreased PNS-SNS activity (decrease PNS, increased SNS, or both), and ultradepressed basal metabolism.

The ELDER parameter alterations suggested that ultradepressed basal metabolic rate related to decreased HRV with aging (*σ* ⇊ ), because decreased PNS-SNS activity (*β*↓) led to increased HRV, rather than decreased HRV (e.g., ref.  ^66^), according to the HRV amplitude indicator (*η*_2_ = *σ*∕*β*).

The increased myocardial electrical resistance coefficient in elderly group during resting was consistent with the previous observations that (i) conductive disorders occurring at the heart conduction beam are usually part of the aging heart^67^; (ii) AP conduction time taken and conduction distance increased proportionally with age; conversely the conduction velocity decreased with age, contributed to loss of Cx43 protein^68^; and (iii) a number of changes with aging do occur in the cardiac conduction system that impact its electrical properties^69^. The decreased PNS-SNS activity could be caused by a decrease in sinoatrial node parasympathetic activity^70,71^ and an increase in sympathetic activity in the heart and vascular system^70^. The ultradecreased change in metabolic rate in elderly group during rest supported the decline in basal metabolic rate with age^71^.

### Congestive heart failure

Comparison between the HR parameters in the YOUNG and CHF groups showed that CHF patients had ultra-elevated myocardial electrical resistance coefficient (*α* ⇈) and mildly increased PNS-SNS activity coefficient (*β*↑) compared to age, which led to elevated HRV stability coefficient (*γ*↑), and ultradepressed standard deviation of myocardial metabolism (*σ* ⇊) leading primarily to ultradepressed HR amplitude indicator (*η*_2_ ⇊).

The ultra-elevated myocardial electrical resistance coefficient supported that T-tubule conductivity is significantly decreased compared with healthy cardiac cells in heart failure^52^. The mildly increased PNS-SNS activity coefficient was consistent with upregulation of the sympathetic overdrive^72^ and abnormal responsiveness of the parasympathetic nervous system in heart failure^73,74^. The ultralow standard deviation of myocardial metabolism supported that myocardial metabolic abnormalities make an important contribution to CHF^75^ but also peripheral tissues and organs^76^.

### ST segment alterations

The ST group had ultralow PNS-SNS activity coefficient (*β* ⇊) resulting in both small HRV stability coefficient (*γ*↓0) and ultrahigh HR amplitude indicator (*η*_2_ ⇈).

The heart rate time series in the ST group had ultrahigh HRV or persistent fluctuation far from equilibrium (see Section 3.1). The ultraincreased HR amplitude indicator predicted ultrahigh HRV amplitude as showed in Figs. 2A and 3. A near-zero stability coefficient predicted persistent fluctuations of HRV far from equilibrium as a nonstationary unit root process. While the ultrahigh HRV or persistent fluctuations far from equilibrium are accompanied by ultralow and very-low-frequency bands of HRV. As shown in Fig. 2A, s20641 with $\widehat{\gamma }=0.0084$ exhibited not only ultrahigh HRV and persistent fluctuations far from equilibrium but also a very low-frequency band. Notably, the most important cause of ST segment elevation is myocardial ischemia so that ST segment elevation is used as a marker of acute myocardial ischemia^77,78^.

These results suggested that ultraelevated HRV in ST segment alterations was related to ultradecreased PNS-SNS activity. A hallmark of cardiovascular disease is thought as cardiac autonomic dysregulation^79^. The phenotype of impaired parasympathetic responsiveness and/or sympathetic hyperactivity due primarily to cardiovascular diseases could result in ultra-decreased PNS-SNS activity. While SNS hyperactivity could be triggered by myocardial ischemia. Myocardial ischemia relates to coronary artery disease (CAD)^80^. CAD-related endothelial dysfunction^81^ may induce a vicious circle of increased SNS activity → vascular wall injury →  development of CAD → myocardial ischemia →  increased SNS activity. Additionally, the ultraderessed PNS-SNS activity coefficient in ST group supported that iatrogenic hyperthyroidism can induce transient ST segment elevation in a patient with normal coronary arteries^82^.

### Ventricular tachycardia/ventricular fibrillation

In contrast with the YOUNG group, we discovered that VT/VF group had ultraelevated myocardial electrical resistance coefficient (*α* ⇈) and ultraelevated PNS-SNS activity coefficient (*β* ⇈) leading to ultraelevated HRV stability coefficient (*γ* ⇈), ultraelevated mean of myocardial metabolism (*ω* ⇈) leading to ultraelevated HR slope indicator (*η*_1_ ⇈), and an ultraelevated standard deviation of myocardial metabolism (*σ* ⇈).

The heartbeat time series in the VT/VF group came from patients with myocardial infarction (63.5%), CAD with no myocardial infarction (28.4%), dilated cardiomyopathy (18.9%), and sustained monomorphic VT (75%) attributed primarily to a chronic infarction scar. A myocardial scar from prior infarct is the most common cause of sustained monomorphic VT in patients with structural heart disease, whereas the most common cause of VF is acute coronary ischemia^83^. The stimulus-driven homeostatic HRV responder accurately resembled most of the HRV time series in the VT/VF group (Fig. 5), which supported the observation that the most common cause of sustained monomorphic VT is a myocardial scar from prior infarct. The VT/VF parameter alterations were consistent with the observation that acute coronary ischemia is the most common cause of VF. CAD is the most common cause of myocardial ischemia, while untreated myocardial ischemia can induce infarction. Acute myocardial ischemia could substantially elevate myocardial resistance^84^ (*α* ⇈) and trigger acute cardiovascular stresses (*ω* ⇈, *σ* ⇈), which in turn could provoke an overactive PNS or underactive SNS (*β* ⇈) condition via a positive correlation between changes in cardiovascular stress (*σ*) and PNS-SNS activity (*β*) (*r* = 0.43, *P* < 0.0005). The ultraelevated PNS-SNS activity coefficient supported that the finding that low-frequency power fell before the onset of VT^85^ and that a hallmark of cardiovascular disease was cardiac autonomic dysregulation^86^.

### Sudden cardiac death

The SCD group had an ultraelevated myocardial electrical resistance coefficient (*α* ⇈), an ultraelevated HR amplitude indicator (*η*_2_ ⇈), and an increased HRV stability coefficient (*γ*↑) due primarily to the ultraelevated myocardial electrical resistance coefficient.

SCD likely occurs when underlying myocardial function cannot respond to elevated changes in the mean of myocardial metabolism under the regulation of PNS-SNS activity in order to keep heart rhythm in an approximate stable range. Thus, the risk factors for SCD should comprise overtly elevated myocardial electrical resistance, overtly elevated standard deviation of myocardial metabolism, and a substantial deviation below or above the approximate HRV stability. We considered the ratios of *α*∕*α*_*c*_, *σ*∕*σ*_*c*_, and *γ*_*c*_∕*γ* or *γ*∕*γ*_*c*_ the measures of these risk factors where the HR parameters *α*_*c*_, *σ*_*c*_, and *γ*_*c*_ of the YOUNG group were control standards for SCD. The probability of multiple independent events happening at the same time equals the multiplication of their individual probabilities. For these reasons, the risk predictor for SCD was introduced by

7 $$\rho =\left\{\begin{array}{ll}\frac{\alpha }{{\alpha }_{c}}\times \frac{\sigma }{{\sigma }_{c}}\times \frac{{\gamma }_{c}}{\gamma } & \,{\rm{i}}{\rm{f}}\,\ \ \gamma \ \le \ {\gamma }_{c}\\ \frac{\alpha }{{\alpha }_{c}}\times \frac{\sigma }{{\sigma }_{c}}\times \frac{\gamma }{{\gamma }_{c}} & \,{\rm{i}}{\rm{f}}\,\ \ \gamma  > {\gamma }_{c}\end{array}\right.$$

The risk predictor for SCD of the VT/VF group was far higher than that of other groups, which confirmed that VT/VF is the first leading cause of SCD. The ST and SCD groups had the second biggest risk predictor for SCD, which was consistent with the fact that ST segment alteration is a leading cause of SCD. The VT/VF and ST groups had the first and second risk predictors for SCD, although they had the biggest and littlest PNS-SNS activity leading to the biggest and littlest HRV stability, respectively. Moreover, our results suggested that SCD could occur in overactive PNS, underactive SNS or overactive SNS conditions^87^.

The risk predictor for SCD could explain why SCD is more likely to occur in the early hours of the morning after awakening than at other times^88^. First, the regulation of HRV stability became weaker (*γ*↓) due to increased SNS activity in the morning upon arising from bed. Second, changes in the myocardial metabolic rate were more likely to reach the highest value (*σ*↑) during the transition from sleep to waking, during which changes of fundamentally different behavioral states occur. According to the risk predictor for SCD, we could predict that SCD was more likely to occur in older men with VT during physical activity than in young healthy women at rest^89^ because of a larger myocardial electrical resistance (*α*↑), larger changes in myocardial metabolic rate (*σ*↑), and a larger deviation above the normal HRV stability (*γ*↑).

## Discussion

In this study, we established the NLARI-HR model and HRV responder as the heartbeat data generative processes driven by myocardial noise or stimulus using the NLARI process based on dynamic analysis. The HRV responder was validated using heartbeat data from 30 healthy subjects and 84 patients. Statistical tests indicated that almost the heartbeat data supported the HRV responder with the stable fixed point (which was called the stable homeostatic HRV responder or homeostatic HRV responder). The homeostatic HRV responder successfully predicted and simulated heartbeat dynamics including long memory for part of dataset, but failed heartbeat data from some ST segment alteration and severe CHF patients. We derived HR physiological parameters from the HR model: the myocardial electrical resistance coefficient, the PNS-SNS activity coefficient (the restoration coefficient), the mean and standard deviation of metabolic disturbances, and wave indicators. When comprising these HR parameters under five different conditions with those for healthy, resting, young subjects, we discovered useful information about heart function, metabolic status, and cardiac autonomic nervous system activity.

The main findings are as follows. (i) The dynamic control mechanism of heart rate was a stable fixed point, rather than a strange attractor or transitions between a fixed point and a limit cycle. However, the stability could collapse into a unit root process as observed in some patients with ST segment alterations and severe CHF. Random walk and Brownian motion are the simple and continuous case of a unit root process. A unit root process is nonstationary and displays a seemingly chaotic behavior but not chaos. The two processes are due to a small and large HR stability coefficient respectively. (ii) HR fluctuation depended on two factors: cardiac disturbances and responses, but not a single factor such as cardiac autonomic modulation. It has been demonstrated that wave slope and amplitude depend on the relative strength of external disturbances to internal responses respectively^36^. Based on the wave indicators, HR slope indicator equaled the ratio of the mean of myocardial disturbances to the myocardial electrical resistance coefficient; HR amplitude indicator equaled the ratio of the standard deviation of myocardial disturbances to the relative strength of PNS against SNS activity (PNS-SNS activity). This implies that when myocardial disturbances remain basically unchanged, HR amplitude is negatively related to PNS-SNS activity. According to this result, PNS activation will decrease HRV amplitude if myocardial metabolic rate remains basically unchanged. However, PNS activation increases HRV amplitude in phenylephrine infusion^90^, suggesting that the infusion would provoke increased myocardial metabolic rate. Myocardial disturbances for a healthy subject usually reflect metabolic demands. When autonomic nervous system activity remains basically unchanged, HR amplitude during resting reflects basal metabolic rate for a healthy subject. Our result could explain why the role of the ANS under resting condition can be elucidated in the low and the high frequency bands (often corresponding to the high and low amplitudes) (e.g., ref. ^2^), but remains unclear under exercise and/or other increased metabolic demands (e.g., ref. ^3^). (iii) Age-related decreases in HRV could be related to age-related decreases in basal metabolic rate; ultrareduced HRV in CHF patients could be related to ultradepressed myocardial metabolism; ultraelevated HRV in ST segment alterations could be related to ultradecreased PNS-SNS activity SNS hyperactivity due to myocardial ischemia; and ultraelevated HR slope in VT/VF patients could be related to acute cardiac stresses, which in turn provoked an overactive PNS or underactive SNS condition. (iv) Ultraderessed PNS-SNS activity could induce ST segment alterations in patients without cardiac artery diseases.

In this study, we compared the restoration coefficient with the classical parametric power spectral analysis to assess the ANS modulation. We showed that the restoration coefficient was positively related to the frequency of a time series in high-frequency bands, whereas the relation did not exist in low-frequency bands. In other words, a low LF/HF ratio could represent high PNS activity, but a high LF/HF ratio did not certainly represent high SNS activity. We confirmed that the classical parametric power spectral analysis was not suitable for non-Gaussian white noise, nonlinear, and nonstationary stochastic processes because the performance of the LF/HF ratio was very nonstationary even in a stationary nonlinear time series (interestingly, an adaptive time scale could significantly reduce the nonstationarity of the LF/HF ratio). In contrast, the parameter sensitivity analysis demonstrated that the performances of the NLARI-HR model with the stable fixed point were robustly stable, while the heartbeat data almost fell in the stable fixed-point range according to our statistical tests.

In summary, the present method provided (i) a plausible explanation for why reduced HRV was associated with ageing and increased mortality risk in patients after myocardial infarction or with advanced heart failure^3,91–93^ by HR wave indicators; (ii) a noninvasive tool for measuring cardiac metabolism and function by the mean and standard deviation of metabolic disturbances; (iii) a noninvasive tool to assess heart health by the myocardial electrical resistance coefficient; and (iv) a stable indicator to assess the ratio between PNS and SNS activity by the restoration coefficient.

The limitation of this method is invalid for the minute scale in describing such as heart rate oscillations linked to vasomotion and modifications of peripheral resistances. The time-scale analysis showed that an adaptive time scale covered by the NLARI-HR model was a few seconds, but a minute scale was never allowed because an over-enlarged time scale can reduce the capability of the HR parameters to extract physiological characteristics from heart rate data. Another limitation is that the NLARI-HR model is unlikely to clarify the intrinsic mechanism of heart rate asymmetry. Time reversibility is one of important properties of linear stationary processes, while time irreversibility is a common signature of nonlinear processes. Temporal asymmetry of short-term heart period variability is an accepted intrinsic property of HRV, which delivers the time irreversibility of HRV as an important nonlinear marker of HRV dynamics^94^. The deterministic system of the NLARI-HR model (in a block of random perturbations) is intrinsic symmetry. The NLARI process is a stochastic process derived by letting the antisymmetric function $f(x)=-\,\alpha x$ express the resistance force for $x=\dot{X}$ and the antisymmetric function $g(x)=-\,\beta x\,\exp (-{x}^{2})$ express the restoration force for *x* = *X* − *μ* where *μ* expresses the equilibrium or average. Thus, the NLARI-HR model has an intrinsic symmetric structure so that this model cannot capture heart rate asymmetry.

In additional, we developed the risk predictor for SCD based on the following assumption: SCD could likely occur in the coexistence of overt-elevated myocardial electrical resistance, overtly elevated myocardial metabolic changes, and a substantial deviation below or above the approximate HRV stability (overactive SNS or overactive PNS conditions). However, we did not offer direct evidence to support the risk predictor for SCD. Although the CHF parameter alterations suggested that CHF was related to subclinical hypothyroidism, empirical evidence is required for the hypothesis. These issues are left to future research.

## Methods

Let Δ*Y*_*t*_ = *Y*_*t*_ − *Y*_*t*−1_. Equation (1) can be rewritten as

8 $$\Delta {Y}_{t}={\theta }_{1}\Delta {Y}_{t-1}+{\theta }_{2}\frac{-{Y}_{t-1}}{\exp ({Y}_{t-1}^{2})}+{\varepsilon }_{t}$$

The HR parameters of Eqs. (1) and (2) were estimated as follows. Because the stable fixed point of the NLARI process is not globally stable^35^, a great stressful perturbation may lead to an inconsistent parameter estimator. Fortunately, our simulation result indicated that decreasing the magnitude of the data could usually avoid the problem. Thus, we let ${X}_{t}={\rm{l}}{\rm{o}}{\rm{g}}\,$(HR or 60/RR). The heart rate measures the number of times the heart beats per minute (bpm). RR (RR interval) is the beat-to-beat variation (second). Hence, HR is just 60/RR. Then, we estimated the parameters of Eq. (8) by OLS using the data ${Y}_{t}={X}_{t}-{ {\hat{a}} }-\widehat{b}t$ where ${\hat{a}}$ and $\widehat{b}$ were given by the OLS regression line *X*_*t*_ = *a* + *bt* + *u*_*t*_ for ${u}_{t} \sim N(0,{\sigma }_{u}^{2})$. We obtained the estimates ${\widehat{\theta }}_{1}$, ${\widehat{\theta }}_{2}$, $\widehat{\sigma }$, $\widehat{\omega }=\widehat{b}(1-{\widehat{\theta }}_{1})$, $\widehat{\alpha }=1-{\widehat{\theta }}_{1}$, $\widehat{\beta }={\widehat{\theta }}_{2}$, ${\widehat{\eta }}_{1}=\widehat{b}$, ${\widehat{\eta }}_{2}=\widehat{\sigma }$/${\widehat{\theta }}_{2}$, and $\widehat{\gamma }=\frac{1}{2}{\widehat{\theta }}_{2}$/$(1+{\widehat{\theta }}_{1})$ for the HRV responder.

The statistical tests, predictions, and simulations in this article were accomplished by the following five codeblocks.

### Estimation

Step 1: Estimate the regression line *X*_*t*_ = *a* + *bt* + *u*_*t*_ by OLS with data ${X}_{t}={\rm{l}}{\rm{o}}{\rm{g}}\,$(HR or 60/RR) to obtain ${ {\hat{a}} }$ and $\widehat{b}$. Let ${Y}_{t}={X}_{t}-{ {\hat{a}} }-\widehat{b}t$.

Step 2: OLS-Estimate Eq. (8) to obtain $\widehat{\theta }={({\widehat{\theta }}_{1}{\widehat{\theta }}_{2})}^{{\prime} }={({Y}^{{\prime} }Y)}^{-1}{Y}^{{\prime} }y$ where

$$\begin{array}{rcc}{Y}^{{\rm{{\prime} }}} & = & ({Y}_{1,0}^{{\rm{{\prime} }}},\,\cdots ,\,{Y}_{1,n-1}^{{\rm{{\prime} }}}),{y}^{{\rm{{\prime} }}}=(\Delta {Y}_{1},\cdots ,\Delta {Y}_{n})\\ {Y}_{1,t-1} & = & (\Delta {Y}_{t-1},-{Y}_{t-1}\exp (-{Y}_{t-1}^{2})),\Delta {Y}_{t}={Y}_{t}-{Y}_{t-1}\,{\rm{f}}{\rm{o}}{\rm{r}}\,t=1,\cdots ,n\end{array}$$

and

$$\begin{array}{ll}{\widehat{\sigma }}_{\widehat{\gamma }} & =\frac{1}{2}\frac{\widehat{\sigma }}{1+{\widehat{\theta }}_{1}}\sqrt{(-2\widehat{\gamma },1){({Y}^{{\prime} }Y)}^{-1}{(-2\widehat{\gamma },1)}^{{\prime} }}\\ \widehat{\sigma } & =\sqrt{\frac{1}{n-2}{(y-Y\widehat{\theta })}^{{\prime} }(y-Y\widehat{\theta })}\\ {s}_{1} & =\widehat{\sigma }\sqrt{(1,0){({Y}^{{\prime} }Y)}^{-1}{(1,0)}^{{\prime} }}\\ {s}_{2} & =\widehat{\sigma }\sqrt{(0,1){({Y}^{{\prime} }Y)}^{-1}{(0,1)}^{{\prime} }}\end{array}$$

### Testing

Step 1: Perform ESTIMATION with sample data $({X}_{t},t=1,\cdots \ ,n)$.

Step 2: Determine whether the 95% confidence intervals ${\hat{\theta }}_{1}\pm {z}_{0.05}{s}_{1}$ and ${\widehat{\theta }}_{2}\pm {z}_{0.05}{s}_{2}$ fall within $(-1,1)$ and $(0,4)$ where *z*_0.05_ is a critical value at the 95% level with *n* − 2 degrees of freedom for two-tailed *t*-tests.

Step 3: Determine whether the *F*-statistic in Eq. ((3)) is greater than the 1% critical value of an *F*-distribution with 1 numerator degrees of freedom and *n* − 2 denominator degrees of freedom.

Step 4: Determine if the *γ*_*n*_-statistic in Eq. (4) is less than  − 3.50 or greater than 11.9 (the 1% critical values for the *γ*_*n*_ distribution in samples *n* = 900 ~ 10000).

If all the results in Steps ${\mathfrak{2}}$ to ${\mathfrak{4}}$ are positive, then the data support ${\theta }_{1}\in (-1,1)$, ${\theta }_{2}\in (0,4)$, $\gamma \in (0,1)$, and *γ* > 0.

### Prediction

Step 1: Samples are divided into in-sample and out-sample: ${\{{X}_{t}\}}_{t=1}^{n}={\{{X}_{t}\}}_{t=1}^{[n/2]}\cup {\{{X}_{t}\}}_{t=[n/2]+1}^{n}$ leading to ${\{{Y}_{t}\}}_{t=[n/2]+1}^{n}$ ($[x]$ is the greatest ingeter of *x*).

Step 2: (In-sample fit): Calculate the 95% confidence intervals ${\widehat{\theta }}_{1,2}\pm {z}_{0.05}{s}_{1,2}$ (${\widehat{\theta }}_{1,2}:{\widehat{\theta }}_{1},{\widehat{\theta }}_{2};{s}_{1,2}:{s}_{1},{s}_{2}$) and $\widehat{\sigma }$ by performing ESTIMATION using ${\{{X}_{t}\}}_{t=1}^{[n/2]}$.

Step 3: (Out-of-sample prediction): Get the output of the HRV responder as close as possible to actual HRV ${\{{Y}_{t}\}}_{t=[n/2]+1}^{n}$ by regulating the parameters ${\theta }_{1,2}\in {\widehat{\theta }}_{1,2}\pm {z}_{0.05}{s}_{1,2}$ (*θ*_1,2_: *θ*_1_, *θ*_2_) for ${\varepsilon }_{t} \sim N(0,{\widehat{\sigma }}^{2})$.

### Simulation

#### Noise-driven HRV responder

Step 1: Calculate the confidence intervals ${\widehat{\theta }}_{1,2}\pm {z}_{0.05}{s}_{1,2}$ and $\widehat{\sigma }$ by performing ESTIMATION using sample data ${\{{X}_{t}\}}_{t=1}^{n}$.

Step 2: Get the output of the HRV responder as close as possible to the actual HRV ${\{{Y}_{t}\}}_{t=1}^{n}$ by regulating ${\theta }_{1,2}\in {\widehat{\theta }}_{1,2}\pm {z}_{0.05}{s}_{1,2}$ for ${\varepsilon }_{t} \sim N(0,{\widehat{\sigma }}^{2})$.

#### Stimulus-driven HRV responder

Step 1: Perform steps 1 and 2 in ESTIMATION to obtain the parameters ${\widehat{\theta }}_{1}$ and ${\widehat{\theta }}_{2}$ using sample data ${\{{X}_{t}\}}_{t=1}^{n}$.

Step 2: Regulate the threshold *c* > 0 to get the output of the HRV responder where ${\theta }_{1,2}={\widehat{\theta }}_{1,2}$ and

9 $${\varepsilon }_{t}={\widehat{\varepsilon }}_{t}=\left\{\begin{array}{ll}c & \,{\rm{i}}{\rm{f}}\,\ {Y}_{t}\ \ge \ c\\ {Y}_{t} & \,{\rm{i}}{\rm{f}}\,\ -c < {Y}_{t} < c\\ -c & \,{\rm{i}}{\rm{f}}\,\ {Y}_{t}\ \le \ -c\end{array}\right.$$

are as close as possible to the actual HRV ${\{{Y}_{t}\}}_{t=1}^{n}$.

### Assessment

Step 1: Perform ESTIMATION to obtain the OLS estimate ${\widehat{\phi }}_{ij}$ of the parameter *ϕ* = *α*, *β*, *ω*, *σ*, *γ*, *η*_1_, *η*_2_ in the *i*-th sample period and the *j-*th subject/patient using sample data $({X}_{ijt},i=1,\cdots \ ,p;j=1,\cdots \ ,q;t=1,\cdots \ ,n)$ for *n* = 900.

Step 2: Calculate the following group-average estimates

10 $$\bar{\phi }=\frac{1}{pq}{\sum }_{i=1}^{p}{\sum }_{j=1}^{q}{\widehat{\phi }}_{ij}$$

and

11 $$sd(\phi )=\sqrt{\frac{1}{pq-1}{\sum }_{i=1}^{p}{\sum }_{j=1}^{q}{({\widehat{\phi }}_{ij}-\bar{\phi })}^{2}}$$

Step 3: Detect the parameter alterations by comparing the HR parameters of the investigated group with the YOUNG group for the group-average estimates.

All the detailed programs used in this article are presented in Supplementary Information.

## Supplementary information

Supplementary information.
