# Supplementary material for: The control mechanisms of heart rate dynamics in a new heart rate nonlinear time series model
Source: Sci Rep. 2020 Mar 16;10:4814. doi: 10.1038/s41598-020-61562-6 (PMC7075874; doi:10.1038/s41598-020-61562-6)
Supplement: Supplementary file 1 — Supplementary information. [file 41598_2020_61562_MOESM1_ESM.pdf]

The control mechanisms of heart rate dynamics in a new heart rate nonlinear time series model

By Zonglu He*
Faculty of Management and Economics
Kaetsu University
2-8-4 Minami-cho, Hanakoganei, Kodaira-shi, Tokyo 187-8578, Japan

December 6, 2019

Abstract

Supplemential Information

Data

Samples: In Fig 2a: chf01 (sample size: 57801–58700), fly02 (1–900), and s20641 (1–900). In Fig 3: 0209.vt1 (1–1000), others (1–1800). In Fig 4: 1–1000. In Fig 5: f1o04 (1–1800). In Fig 2b and Fig 6: YOUNG group (15 subjects): f1y01 to f1y10, f2y01 to f2y05 (1–5400 for each subject, but 1–5013 for f1y07); ELDER group (15 subjects): f1o01 to f1o10, f2o01 to f2o05 (1–5400 for each patient, but 1–4500 for f1o04); CHF group (15 patients): chf01 to chf15 (1–5400 for each patient); ST group (15 patients): s20061, s20081, s20141, s20251, s20401, s20501, s20541, s20621, s20641, s30741 (1–5400); s20261 (12001–17400), s20281 (7001–12400), s20742 (35001–40400), s30742 (20001–25400), s30761 (10001–15400); SCD group: scd30 (1–40500), scd33 (1–40500); and VT/VF group (52 patients): 0003vt1 to 0269vf1 (1-1000).

Source: All data are free available at Fantasia Database
http://physionet.mit.edu/physiobank/database/fantasia/?CachedSimilarAll
for the YOUNG and ELDER groups;
BIDMC Congestive Heart Failure Database
https://physionet.org/physiobank/database/chfdb/
for CHF group;
Long-term ST database
https://www.physionet.org/physiobank/database/ltstdb/
for ST group;
Sudden Cardiac Death Holter Database

*Tel: +81-42-466-3711; E-mail: zongluhe@kaetsu.ac.jp

https://www.physionet.org/physiobank/database/sddb/
for SCD group;
Spontaneous Ventricular Tachyarrythmia Database
https://www.physionet.org/physiobank/database/mvtdb/
for VT/VF group.

Parameters

In Fig 2a, the estimator $\left(\hat{b}, \hat{\theta}_1, \hat{\theta}_2, \hat{\sigma}, \eta_1, \eta_2\right)$ was $(0, -0.2543, 0.1297,\ 0.0058, 0,$ $0.0451)$ for chf01, $(0, 0.2214, 0.4365,\ 0.0257, 0, 0.0630)$ for f1y02; in s20641, $\eta_1 =$ 0.0013 for 131–300, $\eta_1 = -0.0005$ for 301–700, and $\eta_1 = 0.0012$ for 701–900; $\eta_2 = 0.7770$ for 1–900. In Fig 3, the estimator $\left(\hat{\theta}_1, \hat{\theta}_2, \hat{\sigma}\right)$ was $(-0.2086, 0.6061,$ $0.08403)$ for 0209.vt1 in the period 1–500, $(0.0005, 0.2492,\ 0.03580)$ for f1y07 (1–900), $(0.2546, 0.2914,\ \ 0.02051)$ for f1y06 (1–900), $(-0.3018, 0.1258,\ 0.01342)$ for chf06 (1–900), and $(-0.3228,\ 0.3770, 0.00506)$ for chf01 (1–900). In Fig 4, the estimated parameters and the threshold values of $\left(\hat{\theta}_1, \hat{\theta}_2, c\right)$ were given as $(-0.1330, 0.6056, 0.400)$ for 0213.vf1, $(-0.2170,\ 0.6772, 0.200)$ for 0067.vt1, $(0.0505, 1.2747)$ without thresholds for 0261.vt1, $(-0.1354, 0.9483, 0.290)$ for 0209.vf1, $(-0.1159, 0.7625, 0.260)$ for 0040.vt2, and $(-0.3058, 0.6109, 0.150)$ where 0.6109 was the simulated value, rather than the estimate) for 0217.vf1. The predicted HRV in the period 1801–3600 were the output of Eq (2) based on $(-0.2635, 0.1200,\ 0.01241)$ where initial values were the same values as real data. In Fig 5, the estimator $\left(\hat{\theta}_1, \hat{\theta}_2, \hat{\sigma}\right)$ for real heart rate data (f1o04) (1–1800) were given by $\hat{\sigma} = 0.01241$, $\hat{\theta}_1 \pm 1.96 s_1 = -0.2635 \pm 0.0446$ and $\hat{\theta}_2 \pm 1.96 s_2 =$ 0.1136 ± 0.0199 at 5% level. In Fig 6, bar (□) and error bar (|) expressed the group-average estimates and sd from the sample average for all the periods and subjects in each group using n samples over periods 1 to p and subjects 1 to q for each group. In Figs 2b and 6, all of the heartbeat parameters for each group were estimated 90 times; for YOUNG/ELDER/CHF/ST groups: $n = 900$, $p = 6$, and $q = 15$; for SCD group: $n = 900$, $p = 45$, and $q = 2$; for VT/VF group: $n = 1000$, $p = 1$ to 5, and $q = 52$. The correlation coefficient $r = 0.43$ between β and σ for the VT/VF group in the Application section was based on $\hat{\beta}_{ij}$ and $\hat{\sigma}_{ij}$ for $i = 1, 2, \cdots, p$ and $j = 1, 2, \cdots, q$.
